# Facing the pandemic lockdown questionnaire - FPLQ: A brief tool to assess individual differences in front of pandemic experience

**DOI:** 10.1007/s12144-022-02701-2

**Published:** 2022-01-21

**Authors:** Andrea Baroncelli, Lucrezia Tomberli, MariaGiulia Taddei, Enrica Ciucci

**Affiliations:** Department of Education, Languages, Intercultures, Literatures, and Psychology, University of Florence, Via di San Salvi 12, Complesso di San Salvi Padiglione 26, 50135 Florence, Italy

**Keywords:** Pandemic lockdown, Worry, Meaning making, Post-traumatic growth, Social connectedness

## Abstract

This paper presents the development and the initial validation of a self-report questionnaire (the Facing the Pandemic Lockdown Questionnaire - FPLQ) focused on the way in which people faced the impact of the lockdown related to the Coronavirus Disease 19. 504 adults (81.55% females; *M* age = 32.71 years, *SD* = 11.19) took part to the study. Exploratory and confirmatory factor analyses revealed a 15-item 4-factor structure, invariant for gender and age: two dimensions related to maladaptive processes (i.e., “Perception of low social connectedness and lack of routines” and “Health worry”) and two dimensions related to adaptive processes (i.e., “Positive re-thinking” and “Perception of online social connectedness”). Further, we investigated the associations between these dimensions and measures pertaining cognitive (i.e., internal and external health locus of control), emotional (i.e., positive and negative affect), and relational (i.e., attitude and behaviors toward civic engagement) processes, also testing the moderating role of gender and age. Finally, the potential usefulness of this new tool for both extant and future psychological research was highlighted.

## Introduction

The Coronavirus Disease 19 (i.e., Covid-19) is a severe viral acute respiratory syndrome that has caused over 5,000,000 deaths since late 2019 (World Health Organization, 2021). In the early months of 2020, the related virus (i.e., the Severe Acute Respiratory Syndrome Coronavirus 2 - SARS-CoV-2) spread rapidly to a world-wide scale, leading the World Health Organization to declare the pandemic status. Consequently, many countries promulgated the lockdown of the vast majority of activities: millions of citizens broke off their normal daily routines within social contexts, having the permission to leave home only for few activities (e.g., buy food and medicine, and go to work - but limited to sectors deemed essential, such as law enforcement, healthcare, and food industry). As a result, the vast majority of the population had to deal with the breakdown of face-to-face social relationships, the impossibility of carrying out recreational or religious activities in public places, and the urgent need to adapt the methods of study and work through the internet medium (e.g., smart working), with the risk of job loss for all those workers whose work was not possible remotely. In the present research, we focused our attention to the way in which individuals faced the impact of lockdown, and we developed a short questionnaire named Facing the Pandemic Lockdown Questionnaire (FPLQ). Further, we investigated the associations between its dimensions with measures related to emotional, cognitive, and relational processes.

### Facing the pandemic lockdown

The lockdown experienced in the first months of 2020 has represented a non-normative life event and an acute stressor, requiring individuals to undergo a process of reorganization and integration of the ongoing pandemic experience into a coherent life narrative (Brooks et al., 2020; Venuleo et al., 2020). This experience can be considered as a turning point in individuals’ lives that can have potentially changed developmental pathways (McAdams & Bowman, 2001; McAdams et al., 2001). According to McAdams’ perspective, the way in which individuals face and make sense of turning points entails significant transformation in cognitions, affects, and social behaviors towards both the Self and others: in other terms, individuals who face distressing turning point building positive significance show better recovery, higher levels of happiness and wellbeing, and more commitment to generativity (McAdams & Bowman, 2001; McAdams et al., 2001). This is in line with a number of studies showing that individuals who cope with threatening events with a disposition according to which benefits may come out of adversity present an adaptive growth in both intrapersonal and interpersonal processes (Affleck & Tennen, 1996; Taylor et al., 2020; Tedeschi & Calhoun, 2004). The lockdown experience has the characteristics to exert such an impact on individuals’ developmental trajectories. Previous studies on forced mass isolation and quarantine showed the high likeability to develop significant levels of negative emotionality, deficit in emotion regulation, and intrusive and repetitive thoughts leading to anxiety and depression (Bults et al., 2011; Shultz et al., 2016; Thompson et al., 2017). In line with this, recently published papers related to the Covid-19 pandemic have substantially confirmed the above-reported results (e.g., Brooks et al., 2020; Huang & Zhao, 2020). However, the extent of these manifestations can vary significantly from person to person, and sometimes may not even occur. In fact, the same event can result in different processes and in different outcomes among the involved individuals, considering that people do not passively undergo events, but can actively shape their own path of life through their available resources and degrees of agency (Elder, 1998). Therefore, it is essential to investigate the individual differences that characterize the way in which individuals face the lockdown experience, in order to both monitor the ongoing coping processes and possibly guide them through specific interventions. Although we are aware of the multiplicity of both individual and contextual variables that can explain these individual differences, in the present research we focused on measures of cognitive constructs through which people interpret the Covid-19 pandemic and directs subsequent affective, cognitive, and relational processes. To do this, we first reviewed significant constructs concerning adaptive and maladaptive approach in coping with pandemics, and we focused on three thematic areas: 1) a dysfunctional anchoring on extant threats (i.e., the phenomenon of worry), 2) generativity in front of extant threats (i.e., the cognitive processes related to meaning-making and the post-traumatic growth), and 3) a cognitive schema of belongingness concerning enduring perception of closeness with other human being despite of extant threats (i.e., social connectedness).

The development of the FPLQ was realized in the days immediately following the outbreak of the pandemic lockdown in Italy (i.e., March 2020). Following the cognitive-behavioral approach (i.e., stressing that cognitions are the primary determinants in the development and maintenance of adaptive or maladaptive outcomes in response to life events; Kalodner, 2011), we were interested to investigate individual cognitive differences regarding modes of perceiving, thinking, remembering, and problem solving activated to cope with the experience of pandemic. In order to focus our attention on empirically-based salient psychological issues, we performed a literature review of previous findings regarding the impact of pandemics or quarantines. Since the present research was realized with the urgency of measuring ongoing processes whose duration was unknown, we were not able to perform literature review on the basis of robust approaches, such as systematic review or bibliometric analysis (Linnenluecke et al., 2020); nevertheless, we relied on papers of the last two decades that were mainly focused on SARS epidemic (Blendon et al., 2004; Su et al., 2007), H1N1 influenza (Bults et al., 2011; Wang et al., 2011), and Ebola (Rubin et al., 2016; Thompson et al., 2017). In particular, we referred on a rapid review paper by Brooks et al. (2020) that was released at the end of February 2020 and reported the main psychological processes emerged in previous quarantine episodes.

According to results by Brooks et al. (2020), we first paid attention to concern that one’s own life may undergo significant negative changes; for instance, this can be related to health issues (e.g., fear of getting infected, or fear of having to deal with contagion and illness of a loved one), loss of usual routines (e.g., going out, or see friends), or financial problems (e.g., the interruption of professional activities, or a possible global economic crisis). We can argue important individual differences about the strength and the impact of these concerns during the pandemic, ranging from a moderate patterns of activation to particularly high levels that imply worry. Worry is defined as a cognitive phenomenon characterized by repetitive negative thinking: although it is common to respond to threatening events with an appraisal process focused on cognitions related to the anticipation of negative consequences (e.g., “Will I get sick?”, “May I lose my job?”), a pervasive anchoring on these cognitions can lead to worry, that is part of a cognitive attentional syndrome that increases distressing emotions and maintains anxiety and depression, post-traumatic stress disorder, and obsessive-compulsive disorder (Fergus et al., 2013). For this reason, we decided to investigate individual differences in terms of worry related to health, daily habits, and economic aspects in individuals who were facing the pandemic lockdown.

A second area of interest focused on approaching threatens events with an adaptive cognitive processing characterized by high levels of re-elaboration and re-conceptualization. In fact, individuals vary in the degree to which avoid intense fears and concerns on the basis of their ability to undergo a process of meaning making (Boals et al., 2011; McAdams & Bowman, 2001, Singer, 2004). Meaning making is considered central to deal with unexpected critical events related to both death losses (e.g., the passing away of a family member or friend) and non-death losses (e.g., the extreme changes in daily routines and social habits) that cause the lack of reference points used to interpret life events (Neimeyer & Sands, 2011; Roussi & Avdi, 2008). In the present study, we defined meaning-making as a cognitive process that, by persistent efforts over time, make sense of the world and rend it both comprehensible and coherent (Martela & Steger, 2016; Walsh, 2020). Specifically, we were interested to investigate the extent to which individuals facing the lockdown were activated on this adaptive process as a mean to cope with stressors. A very close construct is post traumatic growth: by focusing on positive reinterpretation of their experiences, individuals can emerge from adversities with improved awareness about significant aspects or new possibilities related to their own lives (Tedeschi & Calhoun, 2004). This cognitive process has been associated to positive emotionality and high levels of adaptive coping strategies (Folkman & Moskowitz, 2000; Tedeschi & Calhoun, 2004). Once again, having a tool that identifies how people are activated on these transformative processes can allow to evaluate the strengths and the resources of individuals that are facing pandemic.

Finally, we focused on results by Brooks et al. (2020) indicating that loss of daily routine and reduced contacts with others, due to restrictions imposed by a quarantine, can lead to high levels of boredom and sense of isolation from the rest of the world. In other words, the experience of quarantine can deeply affect the degree to which people perceive they are isolated from others, develop a feeling of loneliness, and perceive social support, which are all central aspects concerning the achievement and maintenance of personal well-being (Lakey & Cohen, 2000; Yu et al., 2020). In the present research, these social aspects were considered within the construct of social connectedness. Social connectedness reflects cognitions of enduring interpersonal closeness with the social world that predispose to experience high levels of positive emotionality and well-being (Lee et al., 2001; Zaki & Williams, 2013). Specifically, arguing that people who face the lockdown with high levels of social connectedness should prevent maladaptive outcomes and promote positive affect, we were interested in investigating individual differences concerning the degree to which individuals perceive closeness and connection with others despite the contingent difficulties of physical distancing. On this regard, we also paid attention to consider a specific aspect of the extant pandemic, consisting in the opportunity - at least for the majority of the population familiar with new technologies - to maintain social relationships thanks to the use of social networks and the participation in online communities.

### The present study

The main aim of the present study was to develop and test psychometric properties of a short questionnaire that assesses individual differences in the way in which adults faced the Covid-19 pandemic lockdown. In so doing, on the basis of the above-presented literature, we considered four different cognitive processes that can be involved in individuals’ coping with threatening events: worry, meaning-making, post-traumatic growth, and social connectedness. Further, the present study also aimed to investigate whether individual differences in the above-mentioned constructs account for differences in a set of cognitive, emotional, and social correlates connected to both adaptive and maladaptive outcomes. As for the cognitive correlates, we considered a measure of health locus of control, that defines individual beliefs about life style and healthy behaviors: people high in internal health locus of control are convinced that health depends on them and are generally involved in more health behaviors, while people high in external locus of control attribute their health to external factors (e.g., significant others, medical staff, God, or the fate) and are more likely to experience depression, anxiety, hopelessness, and learned helplessness (Hovenkamp-Hermelink et al., 2019; Luszczynska & Schwarzer, 2005; Pourhoseinzadeh et al., 2017). As for the emotional correlates, we considered positive and negative state emotionality: the former refers to pleasant engagement (e.g., interested, strong, active) with the actual situation that acts as a protective factor against both anxiety and depression, while the latter is an indicator of emotional distress (i.e., including moods like fear, sadness, guilt, etc.) related to several maladaptive processes, including both anxiety and depression (Clark & Watson, 1991; Terracciano et al., 2003). Finally, considering the social correlates, we focused our attention on civic engagement that refers to the involvement and the commitment in civic activities: individuals with high levels of civic engagement are inclined to higher level of empathy, emotion regulation, and prosocial attitudes (Ahnquist et al., 2012; Bobek et al., 2009; Chan & Mak, 2020; Metzger et al., 2018). We hypothesized that individuals mainly activated on aspects related to worry and/or low social connectedness would most likely incur in maladaptive outcomes (i.e., low levels of perceived control on health issues, high levels of negative emotionality, and poor civic engagement); on the contrary, when meaning making, post-traumatic growth, and/or social connectedness are prevalent, individuals would most likely experience sense of control on own health, less emotional problems, and a greater civic engagement.

Importantly, a recent paper by Varga et al. (2021) examined time-series survey data of more than 200,000 individuals from Western and Northern European countries in order to explore several key mental-health indicators (e.g., loneliness, worries, anxiety, and precautionary behaviors) pertaining the initial lockdown phase (March-July 2020). Their results indicated that some population subgroups showed the poorest mental-health outcomes (e.g., the feeling of loneliness and low perceived social support, both connected to pandemic social isolation, affected more intensely young adults under the age of 30, women, and people with pre-existing chronic conditions), suggesting the need for research to in-depth investigate specific psychological processes of specific subgroups, as well as the urgency for public institutions to develop tailored interventions to prevent negative long-term consequences. Accordingly, in the present paper we paid attention to explore the role of gender and age both in analyzing factor structure of the FPLQ (i.e., by testing measurement invariance) and in exploring the associations between the FPLQ and cognitive, emotional, and social correlates (i.e., by testing interaction terms).

## Materials and methods

### Participants and Procedure

In Italy, some restrictive measures aimed at containing the emerging pandemic were implemented on a local scale, starting from the end of February 2020. The progress of the pandemic led to further restrictive measures to the entire country in the first days of March 2020, with the suspension of educational activities (on March 4) and the implementation of a generalized lockdown to all activities (on March 9). Data collection was realized during the week following the establishment of the national lockdown; it involved a convenience sample, and lasted for 5 days. Participants were voluntaries from Italian background recruited throughout the national territory using social networks and messaging platforms (e.g., Facebook and WhatsApp). Each participant received a link to a Google Form containing the informed consent with privacy policies and other research information. Then, participants who gave their consent received another link to a Google Form containing the questionnaires, and they were asked to respond with reference to the recent pandemic lockdown period. The Google Form was made up by different sections corresponding to the different questionnaires. After a demographic section, the order of questionnaires in the Google Form was the same as they are presented below. At the end of each section, the respondent had to actively move to the next section by clicking on a button: each new section opened with specific instructions for compiling the specific tool. This method can be useful to prevent random answers by anchoring participants' attention to the specific task. Moreover, prior to data analyses, we checked for careless respondents by examining the variance of each participant's responses in each questionnaire. The present sample was made up by 504 adults (81.55% females) ranging in age from 18 to 64 years (*M* age = 32.71 years, *SD* = 11.19). By applying the criteria outlined above about the check for careless respondents, the responses of all participants were considered valid and therefore kept for the subsequent analyses. As for health-related information, 9.52% of the participants indicated to have a chronic disease and/or a compromised immune system. As for information relating to the qualification and job position, 60.52% declared a university degree, and 92.06% declared that they were currently engaged in studying or working activities. Finally, as for their relationship status, 41.07% of the participants reported they were engaged in a sentimental relationship with cohabitation, 29.96% was not in a romantic relationship at the time of data collection, and 28.97% was engaged in a sentimental relationship without cohabitation.

### Measures

#### The facing the pandemic lockdown questionnaire - FPLQ

The Facing the Pandemic Lockdown Questionnaire - FPLQ is a short and handy tool to assess cognitive processes activated in response to the Covid-19 pandemic lockdown. The item-generation procedure was based on a review of the psychological published papers on pandemic and mass isolation (see the above-reported literature). This procedure was first conducted by three of the present authors, and an initial pool of items were reviewed and discussed with four additional experts in the field of psychological research and clinical practice, leading to the final version of the FPLQ. Specifically, the worry items (n = 7) combined attention to health, economic-working aspects, and daily habits. The meaning-making (n = 3) and post-traumatic growth (n = 3) items focused on in-depth thinking and rediscovering of significant personal and social aspects about habits and values. Finally, the social connectedness items (n = 5) investigated the perception to be isolated and alone, along with the perceived social support that can derive from online relationships (see Table 1). Each item was posed on a 5-point Likert-type scale (from 1 = “never” to 5 = “always”).

**Table 1:** Factor loadings from exploratory factor analysis (n = 252).

Item	Content	Theoretical construct	Factor loadings			
	With reference to the lockdown caused by the current health situation,... *[Con riferimento al lockdown causato dalla situazione sanitaria attuale,…]*		Factor 1: Perception of low social connectedness and lack of routines	Factor 2: Positive re-thinking	Factor 3: Health worry	Factor 4: Perception of online social connectedness
1	I am worried about the economic crisis. *[mi preoccupa la crisi economica.]*	Worry	.26	.03	.09	.14
2	I am rediscovering passions that I had overlooked. *[sto riscoprendo passioni che avevo messo da parte.]*	Post-traumatic growth	-.14	**.87**	-.07	-.02
3	I am thinking about how much I like/I don't like my job. *[sto riflettendo su quanto mi piaccia/non mi piaccia il mio lavoro.]*	Meaning making	.11	**.41**	.03	-.01
4	I am feeling the need to return to normality. *[sento il bisogno di tornare alla normalità.]*	Social connectedness	**.70**	.05	.08	-.01
5	I am worried about the thought of getting sick. *[mi preoccupa il pensiero di ammalarmi.]*	Worry	-.11	-.13	**.96**	.02
6	I am rediscovering old ways of spending time (e.g., playing cards, board games). *[sto riscoprendo vecchi modi di passare il tempo (ad es., giocare a carte, giochi da tavolo).]*	Post-traumatic growth	-.12	**.43**	.02	.28
7	I am thinking about how important it is for me to spend time with people. *[sto riflettendo su quanto sia importante per me passare il tempo con le persone.]*	Meaning making	.46	.37	.06	.06
8	I am feeling part of an online community (e.g., Instagram, Social Networks). *[mi sto sentendo parte di una community online (ad es., Instagram, Social Networks).]*	Social connectedness	.04	.10	-.08	**.61**
9	I am worried about the thought that my loved ones could get sick. *[mi preoccupa il pensiero che si ammali un mio caro.]*	Worry	-.02	.13	**.46**	.05
10	I am rediscovering the time to do many things that I had left aside. *[sto riscoprendo il tempo per fare molte cose che avevo lasciato da parte.]*	Post-traumatic growth	-.11	**.81**	-.04	-.08
11	I am thinking about what things really matter to me. *[sto riflettendo su quali siano le cose che contano davvero per me.]*	Meaning making	.32	**.51**	.14	-.01
12	I am feeling supported by influencers and people around the world. *[mi sto sentendo sostenuto da influencer e da persone in tutto il mondo.]*	Social connectedness	-.01	-.11	.05	**.91**
13	I am worried about the thought that I may die. *[mi preoccupa il pensiero di poter morire.]*	Worry	.07	.02	**.76**	-.08
14	I am feeling more alone than usual. *[mi sto sentendo più solo del solito.]*	Social connectedness	**.62**	.02	.01	-.09
15	I am worried about not being able to go out and do the things I usually do. *[mi preoccupa non poter uscire e fare le cose che faccio di solito.]*	Worry	**.82**	-.14	-.06	-.004
16	I am feeling caged. *[mi sento ingabbiato.]*	Social connectedness	**.85**	-.11	-.06	-.03
17	I am worried about the negative consequences on my work. *[mi preoccupo che il mio lavoro possa venire compromesso.]*	Worry	.27	.10	-.07	.16
18	I am worried not to see friends and/or companions anymore. *[mi preoccupa non vedere più amici e/o compagni.]*	Worry	**.71**	-.09	-.05	.06
	***McDonald’s Omega***		***.84***	***.75***	***.80***	***.59*** ^***+***^
	***Factor Correlations***					
	Factor 1		-			
	Factor 2		.36	-		
	Factor 3		.27	.35	-	
	Factor 4		.31	.49	.23	-

Factor loadings in bold indicate to which factor each item was attributed. Items 1, 7, and 17 did not meet retention criteria. ^+^ Since this scale is composed by two items, reported values refers to correlational coefficients

#### Health locus of control

The Health Locus of Control Scale - HLCS (Donizzetti & Petrillo, 2015) was adopted to assess individual differences in health locus of control. It is a self-report questionnaire with 13 items on a 5-point Likert-type scale (from 1= “strongly disagree” to 5 = “strongly agree”) that load on three dimensions: internal health locus of control (8 items, e.g., “I am the only one responsible for my health”, McDonald’s Omega in the present sample = .88), other health locus of control (3 items, e.g., “My physical health depends on my closest friends”, McDonald’s Omega in the present sample = .67), and God health locus of control (2 items, e.g., “If God wants, my physical health can improve”, correlation coefficient between the two items in the present sample = .48).

#### State emotionality

State emotionality related to the Covid-19 pandemic was assessed using the Positive and Negative Affect Schedule - PANAS (Watson et al., 1988; Italian version by Terracciano et al., 2003), a self-report questionnaire consisting of two 20-item scales to measure both positive affect (10 item, e.g., “Strong”, “Inspired”, McDonald’s Omega in the present sample = .86) and negative affect (10 item, e.g., “Afraid”, “Upset”, McDonald’s Omega in the present sample = .88) in relation to the ongoing pandemic lockdown. Each item was rated on a 5-point Likert-type scale (from 1 = “lightly” to 5 = “extremely”).

#### Civic engagement

The level of civic engagement was assessed with the Civic Engagement Scale - CES (Doolittle & Faul, 2013), a self-report questionnaire consisting of 14 items. This tool measures two specific aspects of engagement: civic attitudes (i.e., the beliefs and feelings about personal involvement and perceived utility for the community; 8 items evaluated on a 7-point Likert-type scale from 1= “disagree” to 7 = “agree”, e.g., “I feel responsible for my community”; McDonald’s Omega in the present sample = .85) and civic behaviors (i.e., the actions realized for the benefit of the community; 6 items evaluated on a 7-point Likert-type scale from 1= “never” to 7 = “always”, e.g., “I help members of my community”; McDonald’s Omega in the present sample = .83).

### Data analyses

- *Step 1: Exploratory and confirmatory factor analyses on items from the FPLQ*

First of all, the sample was randomly divided in two subsamples of 252 participants. The first subsample was used to perform an exploratory factor analysis (EFA; subsample#EFA: females = 206; *M* age = 32.68 years, *SD* = 11.20) and the second subsample was employed to realize a confirmatory factor analysis (CFA; subsample#CFA: females = 205; *M* age = 32.73 years, *SD* = 11.21).

The EFA approach was applied to the 18 observed items using the IBM SPSS Statistics 26 program (IBM Corp., 2019). The adequacy of our data was evaluated exploring the form of distribution of each item using the indices of skewness and kurtosis (scores ranging between −2.00 and +2.00 indicate a normal distribution; George & Mallery, 2010), verifying whether there was a significant number of factors in the data using the Kaiser-Meyer-Olkin’s sampling adequacy criteria (i.e., KMO; values lower than .50 are considered unacceptable - Kaiser, 1974), and testing the hypothesis that correlations between variables were greater than expected by chance by the mean of the Bartlett’s sphericity test (in this, the *p*-value must be significant - Bartlett, 1950). As suggested by Osborne (2014), to determine the number of factors to extract, we combined the theory-driven approach (that consists in extracting a number of factor equal to what is expected following the theory that has driven the development of the questionnaire), the examination of the scree diagram (i.e., obtained computing the diagram of the eigenvalues, that represents how much of the variance of the observed variables each factor explains, and observing the number of points that are above the point of inflexion in the diagram), the Kaiser criterion (that suggests to retain all factors that have an eigenvalue higher than 1.00), and the technique of parallel analysis (that compares eigenvalues from the EFA with eigenvalues from randomly generated uncorrelated data, and suggests to retain factors with eigenvalues that are greater than the eigenvalues from the random data). The EFA was performed using the principal axis method in order to avoid distortions due to data distributions, and a promax rotation allowed for correlations between latent factors; only items with a primary factor loading greater than |.40| and without cross loadings greater than .32 were retained (Costello & Osborne, 2005). Internal consistency of each emerged factor was inspected using McDonald’s Omega. McDonald Omega has been proved to be a more sensible index of internal consistency compared to more traditional indices (e.g., Cronbach’s Alpha; Dunn et al., 2014); although there is no consensus on a cutoff deemed sufficient, most of empirical studies have indicated .60 or .70 as a minimum standard of reliability (Cho & Kim, 2015). Moreover, Cho and Kim (2015) warn about extremely high values of reliability, as they could indicate item redundancy. Two items are redundant when they do not add new information, and we tested this for each emerged factor using inter-item correlations (i.e., the correlations between one item and all other items in the same factor); according to DeVellis (2012), values above .70 suggest redundancy and the need to re-consider the simultaneous presence of both items within that dimension.

The CFA approach was realized using the R Package lavaan (Rosseel, 2012). Once again, we explored the form of distribution of each item, and, in order to avoid distortions due to data distribution, we used the robust variant of the maximum likelihood (MLR) as estimator. For first, we tested the theory-driven model with all the 18 items (Model 1). After that, we tested three increasingly complex models with the 15 items retained after the EFA: a one-factor model in which all items load onto a single general factor (Model 2); a two-factor model in which items referring to a maladaptive cognitive phenomenon (i.e., worry) load onto a single factor, while items referring to adaptive cognitive processes (i.e., post-traumatic growth, meaning making, and social connectedness) load onto a different single factor (Model 3); a four-factor model reflecting the factor structure emerged by the EFA (Model 4); finally, possible modification indices were considered (Model 5). Due to the high sensitivity of the χ^2^ index to sample size, goodness of fit of each model was evaluated using the robust versions of the comparative fit index (CFI), the robust versions of the Tucker-Lewis index (TLI), the robust versions of the root mean square error of approximation (RMSEA), and the standardized root mean square residual (SRMR): both CFI and TLI measure relative fit compared to the null model, and they should exceed .95; RMSEA and SRMR are measures of absolute model fit, and they are considered acceptable if lower than .08 (Hooper et al., 2008). In addition, inter-factor correlations (i.e., φ) were computed.

- *Step 2: Measurement invariances of FPLQ factors considering gender and age*

Measurement invariances related to gender (i.e., males vs females) and age (i.e., over vs below mean age) were investigated within the full sample applying two multi-group confirmatory factor analysis procedures with the R Package lavaan (Rosseel, 2012): configural invariance (i.e., the baseline model) tests whether, without constrains on parameters, the same items measure the same factors across groups. Metric invariance tests whether factor loadings are equivalent across groups. Scalar invariance tests for the equivalence of the intercepts. Finally, strict invariance tests whether the indicators’ residual variances are equal across groups. According to Chen (2007), we avoided the use of the χ^2^ difference test due to its sensitivity to sample size and normality of data distribution, and the increasing complexity of invariance levels was evaluated considering differences in CFI values ≤ - .010 along with differences in RMSEA values ≥ .015 as indicators of lack of measurement invariance.

- *Step 3: Descriptive statistics and regression analyses concerning the FPLQ and the other study variables*

Descriptive statistics of both emerged factors and other study variables were calculated (i.e., mean, standard deviation, skewness and kurtosis), along with zero-order correlations (i.e., Pearson’s *r*); moreover, *t*-tests for gender differences regarding emerged factor were calculated. To evaluate the unique contribution of each emerged scale of the FPLQ along with the potential moderating role of both gender and age to the other study variables (i.e., state emotionality, health locus of control, and civic engagement), linear hierarchical regression analyses were performed. Although in a cross-sectional study the directionality of the associations between the variables cannot be established, the application of a regression approach requires assumptions about which variables to consider as independent variables and which variables to consider as criterion variables. Guided by the theoretical framework outlined in the introduction, the present analyses assumed that the way in which individuals face the Covid-19 pandemic lockdown can influence the development of specific internal or external control beliefs on own pandemic-related health situation, individuals’ state emotionality, and the propensity to civic engagement during the pandemic period. As a consequence, gender and age were entered along with all scales of the FPLQ in Step 1, followed by the interaction terms between the four scales of the FPLQ and gender in Step 2a; gender was replaced with age in Step 2b. Scores for age and for the scales of the FPLQ were mean-centered for purposes of these analyses. Given our study’s sizable total sample size, only associations of at least modest effect size (*r* or *β* ≥ .20, with *p* < .001) were emphasized in the text to highlight the findings most likely to be meaningful and replicable; moreover, the significant interaction terms in Step 2 (i.e., either in Step 2a or in Step 2b) were reported only if their exploration conducted according to Holmbeck’s (2002) procedure resulted in evidences at least of modest effect size (β ≥ .20, with *p* < .001).

## Results

- *Step 1: Exploratory and confirmatory factor analyses on items from the FPLQ*

Considering the 18 items of the FPLQ, skewness scores ranged between -1.11 and .81, and kurtosis scores ranged between -1.38 and .61. The results from our dataset indicated that the KMO index was middling (.79). The result of Bartlett’s sphericity test was *χ*^2^ = 1696.41, *df* = 153, *p* < .001. The scree-diagram suggested to extract five factors (that corresponded to those with eigenvalues greater than 1.00; 64.62% of explained variance). Nevertheless, considering that this solution resulted in two factors with only two items, and according both to the four-factor theory-driven model and to the parallel analysis indicating that four factors presented eigenvalues greater than the eigenvalues from the random data, we decided to extract a four-factor structure solution (58.20% of explained variance). Table 1 includes rotated factor loadings: items 1, 7 and 17 failed to reach the retention criteria, and they were excluded from subsequent analyses. To summarize, factor 1 included 5 items reflecting the worry related to a perception of social isolation and feelings of loneliness, and it was labeled “Perception of low social connectedness and lack of routines”. It accounted for 27.99% of variance and its factor loadings ranged from |.62| to |.85|; the McDonald’s Omega for this subscale was .84, and values of inter-item correlations did not exceed .63. Factor 2, labeled “Positive re-thinking*”*, comprised 5 items relating to post-traumatic growth and meaning making. It accounted for 13.34% of variance and its factor loadings ranged from |.41| to |.87|; the McDonald’s Omega was .75, and values of inter-item correlations did not exceed .64. Factor 3, labeled “Health worry*”* consisted of 3 items reflecting the worry about one’s own and loved ones’ health; it accounted for 9.66% of variance and its factor loadings ranged from |.46| to |.96|. The McDonald’s Omega for this subscale was .80, and values of inter-item correlations did not exceed .69. Lastly, Factor 4 comprised 2 items relating to the perception of social connectedness and support through the online social media, and it was labeled “Perception of online social connectedness*”*. It accounted for an additional 7.21% of variance and its factor loadings were .61 and .91. The correlation coefficient between the two items of this scale was .59.

**Fig. 1:**
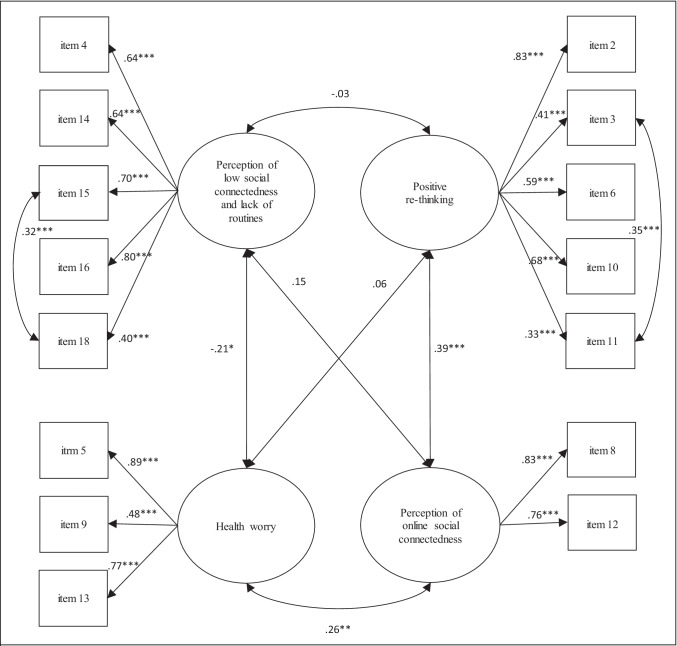
Standardized factor loadings and correlation indices from confirmatory factor analysis (n = 252). Notes. * *p* < .05, ** *p* < .01, *** *p* < .001. McDonald’s Omegas were .79 for perception of low social connectedness and lack of routines, .70 for Positive re-thinking, .80 for health worry, and correlation between the two items of Perception of online social connectedness was .63

Fit indices concerning CFA were reported in Table 2; standardized factor loadings and correlation indices were reported in Figure 1. The goodness of-fit indices for Model 1 were not sufficient. Also Model 2 and Model 3 showed unsatisfactory fit indices. Model 4 showed better fit indices, that improved in Model 5 when two specific modification indices (between the items “I am worried about not being able to go out and do the things I usually do” and “I am worried not to see friends and/or companions anymore” from the “Perception of low social connectedness and lack of routines” factor, i.e., 19.858, and between “I am thinking about how much I like/I don't like my job” and “I am thinking about what things really matter to me” from the “Positive re-thinking” factor, i.e., 29.342) were considered.

- *Step 2: Measurement invariances of the FPLQ factors considering gender and age*

**Table 2:** Comparison of different factor models for CFA

Model	χ^2^	*df*	*p*	CFI	TLI	RMSEA [95% CI]	SRMR
Model 1 (theory-driven four-factor model)	596.161	129	< .001	.623	.553	.121 [.111;.131]	.116
Model 2 (one-factor model)	772.891	90	< .001	.347	.239	.174 [.162;.185]	.159
Model 3 (two-factor model)	511.390	89	< .001	.596	.523	.137 [.126;.149]	.117
Model 4 (four-factor model)	197.794	84	< .001	.888	.860	.074 [.061;.088]	.074
Model 5 (four-factot model with modification indices)	148.836	82	< .001	.934	.916	.058 [.043;.072]	.071

CFI = comparative fit index, TLI = Tucker Lewis index, RMSEA = root mean square error of approximation, SRMR = standardized root mean square residual

Table 3 reported the model fits concerning the measurement invariance analyses. Considering that the cut-off criteria indicated by Chen (2007) were not exceeded in any step, strict measurement invariance was assumed for both gender and age.

- *Step 3: Descriptive statistics and regression analyses concerning the FPLQ and the other study variables*

**Table 3:** Comparison of different factor models testing measurement invariance.

Model	χ^2^	*df*	*p*	CFI	RMSEA	ΔCFI	ΔRMSEA
*Gender*							
Configural	320.208	164	< .001	.928	.063	-	-
Metric	337.952	175	< .001	.925	.062	-.003	-.001
Scalar	362.512	186	< .001	.919	.063	-.006	.001
Strict	389.243	201	< .001	.913	.062	-.006	-.001
*Age*							
Configural	338.663	164	< .001	.920	.066	-	-
Metric	344.804	175	< .001	.922	.063	.002	-.003
Scalar	373.662	186	< .001	.914	.065	-.008	.002
Strict	390.553	201	< .001	.914	.062	.000	-.003

CFI = comparative fit index, RMSEA = root mean square error of approximation

Descriptive statistics and zero-order correlations (i.e., Pearson’s *r*) between all study variables were reported in Table 4. Results of regression analyses were reported in Table 5. All variables were normally distributed; as for dimensions of the FPLQ, they presented weak and positive correlations, with the exception of the positive association between positive re-thinking and perception of online social connectedness (*r* = .39, *p* < .001). Moreover, there was a negative association between age and perception of online social connectedness (*r* = -.26, *p* < .001). Further, females compared to males presented higher scores in positive re-thinking (*t* test = 4.75, *p* < .001, Cohen’s *d* = .55; *M* females = 3.19, *DS* = .85, *M* males = 2.71, *DS* = .93) and perception of online social connectedness (*t* test = 4.15, *p* < .001, Cohen’s *d* = .48; *M* females = 2.52, *DS* = 1.18, *M* males = 1.96, *DS* = 1.10).

**Table 4:** Descriptive statistics and zero-order correltions (Pearson’s *r*) for study variables

Variable	McDonald’s Omega	*M* (*DS*)	Skewness (*SE*)	Kurtosis (*SE*)	1	2	3	4	5	6	7	8	9	10	11
1 FPLQ-Perception of low social connectedness and lack of routines	.81	3.26 (.95)	.01 (.11)	-.85 (.22)	/										
2 FPLQ-Positive re-thinking	.73	3.10 (.88)	-.16 (.11)	-.39 (.22)	.16***	/									
3 FPLQ-Health worry	.80	3.36 (.86)	.06 (.11)	-.69 (.22)	.18***	.18***	/								
4 FPLQ-Perception of online social connectedness	.61^+^	2.42 (1.19)	.50 (.11)	-.79 (.22)	.19***	.39***	.18***	/							
5 HLCS-Internal	.88	3.95 (.68)	-.34 (.11)	-.26 (.22)	.13**	.11*	.01	.002	/						
6 HLCS-External (Others)	.67	2.02 (.81)	.63 (.11)	-.17 (.22)	.32***	.21***	.18***	.26***	-.14**	/					
7 HLCS-External (God)	.48^+^	2.06 (1.10)	.71 (.11)	-.43 (.22)	.12**	.12**	.16***	.13**	.10*	.17***	/				
8 PANAS-PA	.86	3.25 (.76)	-.18 (.11)	.01 (.22)	-.22***	.12**	-.002	.06	.12**	-.03	.12**	/			
9 PANAS-NA	.88	2.60 (.83)	.22 (.11)	-.58 (.22)	.39***	.09*	.40***	.19***	-.01	.27***	.10*	-.35***	/		
10 CES-Attitude	.85	5.36 (.98)	-.70 (.11)	.52 (.22)	-.01	.31***	.17***	.29***	.02	.15***	.09*	.17***	.04	/	
11 CES-Behaviors	.83	4.32 (1.32)	-.08 (.11)	-.46 (.22)	-.02	.20***	-.01	.18***	-.08	.13**	.04	.23***	-.05	.68***	/

FPLQ: Facing the Pandemic Lockdown Questionnaire. PANAS: Positive and Negative Affect Schedule. HLCS: Health Locus of Control Scale. CES: Civic Engagement Scale.

+ Since this scale is composed by two items, reported values refers to correlational coefficients. *p < .05, **p < .01, ***p < .001

**Table 5:** Regression analyses testing the unique role of gender, age, and FPLQ scales on the other study variables

	Gender	Age	FPLQ-Perception of low social connectedness and lack of routines	FPLQ-Positive re-thinking	FPLQ-Health worry	FPLQ-Perception of online social connectedness	*F*	*R*^*2*^
HLCS-Internal	.15**	-.01	.12**	.14***	-.01	-.05	(6,503) = 4.467***	.04
HLCS-External (Others)	.03	-.04	.25***	.10*	.09*	.15** (a)	(6,503) = 15.618***	.15
HLCS-External (God)	.05	.12**	.08	.08	.11*	.10*	(6,503) = 5.233***	.05
PANAS-PA	.24***	.16***	-.25*** (b)	.18***	-.01	.13**	(6,503) = 15.285***	.15
PANAS-NA	-.13**	-.08	.32***	-.08	.34***	.05	(6,503) = 34.723***	.29
CES-Attitude	-.06	.09*	-.09*	.22*** (c)	.09*	.22***	(6,503) = 15.286***	.15
CES-Behaviors	-.06	.06	-.06	.16*** (d )	-.06	.15**	(6,503) = 6.095***	.06

FPLQ: Facing the Pandemic Lockdown Questionnaire. PANAS: Positive and Negative Affect Schedule. HLCS: Health Locus of Control Scale. CES: Civic Engagement Scale. *p < .05, **p < .01, ***p < .001

(a) There was a significant two-way interaction effect for perception of online social connectedness and Age (β =.13, *p *< .01; F(10,503) = 11.265, *p *< .001; ΔR^2^ = .02, *p* < .01, R^2^ = .17): β = .34, *p* < .001 in older; β = .03, *p* > .05 in younger

(b) There was a significant two-way interaction effect for perception of low social connectedness and lack of routines and Gender (β =.14, *p* < .01; F(10,503) = 10.382, *p* < .001; ΔR^2^ = .01, *p* < .05, R^2^ = .16): β = -.30, *p* < .001 in females; β = .001, *p* > .05 in males

(c) There was a significant two-way interaction effect for positive re-thinking and Age (β = -.13, *p* < .01; F(10,503) = 10.490, *p* < .001; ΔR^2^ = .01, *p* < .05, R^2^ = .16): β = .10, *p* > .05 in older; β = .35, *p* < .001 in younger

(d) There was a significant two-way interaction effect for positive re-thinking and Age (β = -.20, *p* < .001; F(10,503) = 5.971, *p* < .001; ΔR^2^ = .03, *p* < .001, R^2^ = .09): β = -.02, *p* > .05 in older; β = .36, *p* < .001 in younger

**Fig. 2:**
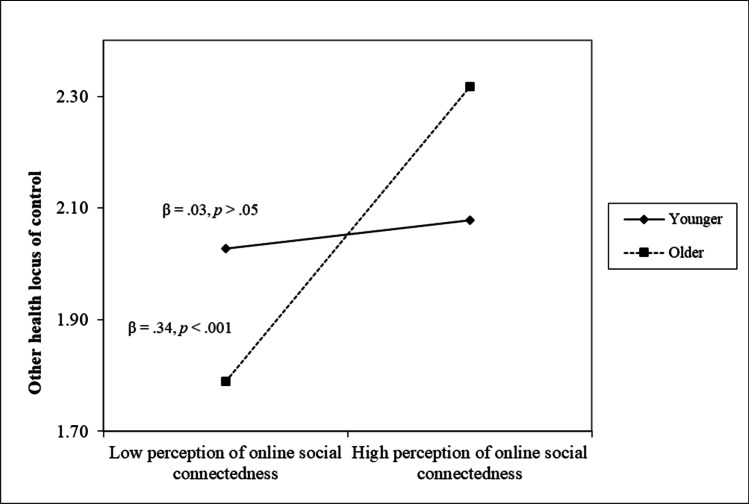
The moderating role of age in the association between perception of online social connectedness and other health locus of control

As for health locus of control during the pandemic, correlation analyses indicated that higher scores in perception of low social connectedness and lack of routines, positive re-thinking, and perception of online social connectedness were related to higher levels of external (i.e., other people) locus of control (*r*s = .32, .21, and .26, *p* < .001, respectively). Nevertheless, regression analyses highlighted the unique positive role of perception of low social connectedness and lack of routines in the full sample (*β* = .25, *p* < .001) and the unique positive role of perception of online social connectedness only for older participants (*β* = .34, *p* < .001 for older, *β* = .03, *p* > .05 for younger, see Fig. 2) in the associations to external health locus of control related to other people. No associations emerged considering neither internal health locus of control, nor external health locus of control related to God.

**Fig. 3:**
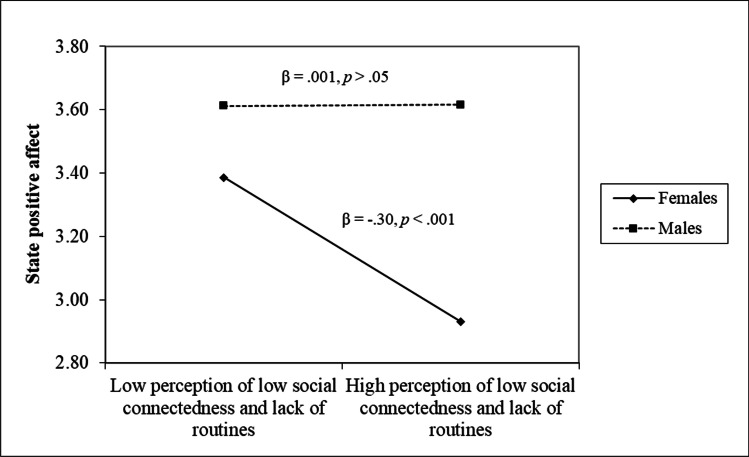
The moderating role of gender in the association between perception of low social connectedness and lack of routines and state positive affect

Considering the associations between FPLQ scales and the state emotionality variables, a negative association between perception of low social connectedness and lack of routines and positive affect at both zero-order (*r* = -.22, *p* < .001.) and regression (*β* = -.25, *p* < .001) levels emerged; this evidence was qualified by a significant interaction term with gender (see Fig. 3), indicating that higher levels of perception of low social connectedness and lack of routines were related to lower levels of positive affect in females (*β* = -.30, *p* < .001) but not in males (*β* = .001, *p* > .05). Moreover, perception of low social connectedness and lack of routines and health worry were uniquely and positively associated to state negative affect (*r*s = .39 and .40, *p* < .001; *β*s = .32 and .34, *p* < .001, respectively).

**Fig. 4:**
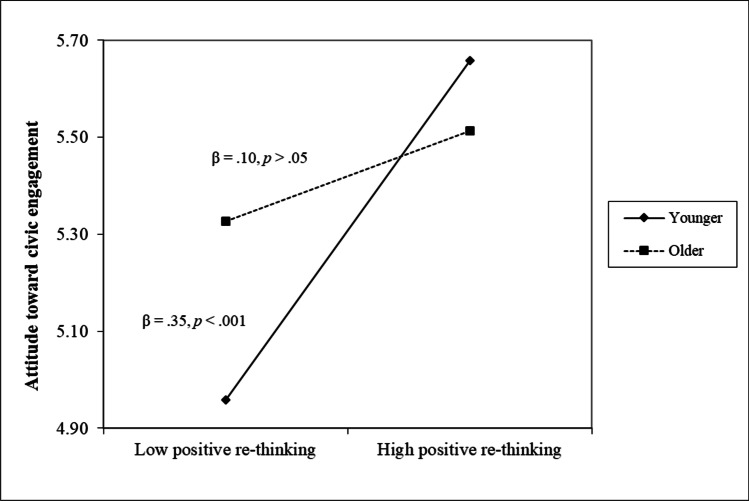
The moderating role of age in the association between positive re-thinking and attitude toward civic engagement

**Fig. 5:**
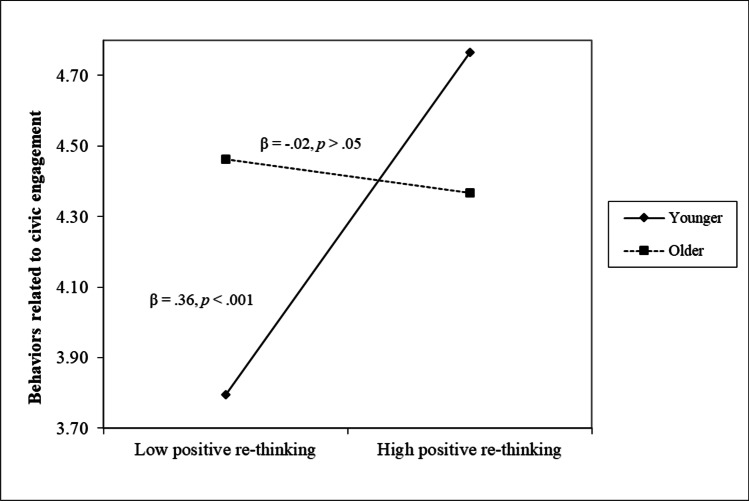
The moderating role of age in the association between positive re-thinking and behaviors related to civic engagement

Lastly, positive re-thinking and perception of online social connectedness resulted to be positively correlated to the attitude toward civic engagement (*r*s = .31 and .29, *p* < .001, respectively), and positive re-thinking was also positively correlated to behaviors related to civic engagement (*r* = .20, *p* < .001). Regression analyses clarified that both positive re-thinking and perception of online social connectedness were uniquely associated to higher levels of attitude toward civic engagement (both *β*s = .22, *p* < .001), even if two significant interaction terms between positive re-thinking and age (see Fig. 4 and Fig. 5) further clarified that higher levels of positive re-thinking were positively associated to both attitude and behaviors pertaining civic engagement in younger participants (*β*s = .35 and .36, *p* < .001, respectively) rather than in older participants (*β*s = .10 and -.02, *p* > .05, respectively).

## Discussion

The present study was realized in order to develop a short and handy questionnaire to assess the way in which adults faced the Covid-19 pandemic lockdown, the Facing the Pandemic Lockdown Questionnaire - FPLQ. The questionnaire was developed and administered in the days immediately following the outbreak of the pandemic lockdown in Italy (i.e., March 2020), and it was based on empirically salient psychological issues emerged by a review of literature on previous pandemics or quarantines. We moved within a cognitive-behavioral theoretical framework, that led us to focus on individual differences regarding cognitive styles activated to cope with the experience of pandemic. The 18 items making up the FPLQ were subjected to an exploratory and confirmatory factor analysis approach. Results indicated a four-factor structure invariant for gender and age, with two factors - “Perception of low social connectedness and lack of routines” and “Health worry” - that refer to maladjustment, and two factors - “Positive re-thinking” and “Perception of online social connectedness” - that refer to adjustment. All factors presented unique associations with a series of criterion variables.

As for factors related to maladjustment, “Perception of low social connectedness and lack of routines” contains items related to a pervasive anchoring on cognitions of caging and impotence. Individuals high in this factor were also high in scores of external health locus of control, as well as in scores of negative affect. Considering both previous literature in health locus of control (Hovenkamp-Hermelink et al., 2019; Luszczynska & Schwarzer, 2005; Pourhoseinzadeh et al., 2017) and the tripartite model of anxiety and depression (Clark & Watson, 1991), these correlates are typical in the context of both anxiety and depression symptoms. As a results, this FPLQ subscale could be useful to intercept subjects particularly weakened by the pandemic, who are experiencing a situation of loneliness and great psychological distress or psychopathology. Moreover, “Perception of low social connectedness and lack of routines” was also negatively related to positive affect (i.e., a specific indicator of depression, Clark & Watson, 1991) within the female subsample; in line with the gender differences found by Varga et al. (2021), women would be at higher risk than men to develop a depressive symptomatology when disruption of social connections and routines are perceived as intense and pervasive. Another factor (i.e., “Health worry”) refers to a disposition of the Self toward pervasive anchoring on negative thoughts and feelings related to both own and others’ health. This factor presented a positive association to negative affectivity, and this was in line with the theoretic framework that places the worry construct in the context of an attentional syndrome that increases distressing emotions and maintains anxiety and depression (Fergus et al., 2013). This FPLQ subscale could be useful to monitor health-related concerns about the pandemic, allowing to differentiate individuals with normative levels (that, even if emotionally unpleasant, activate protective strategies) from individuals with high levels that could lead to anxious or depressive states. This appears particularly important, considering a growing body of research on extant pandemic, highlighting the importance to consider the role of Covid-19 related worries in order to manage disease and distress (Boyraz et al., 2020; Grossman et al., 2021; Taylor et al., 2020).

As for factors related to adjustment, the factor named “Positive re-thinking” represents a cognitive style related to meaning making and post-traumatic growth, both aspects that have emerged as protective factors in the current pandemic (Chen et al., 2021; Milman et al., 2020; Tomaszek & Muchacka-Cymerman, 2020; Vazquez et al., 2021; Walsh, 2020). Specifically, this dimension was positively associated to levels of civic engagement: individuals who faced the restrictive measures of pandemic with the proneness to observe themselves and (re)discover salient dimensions connected with their authenticity showed higher disposition to generatively open to others and to society, even in the context of physical distancing. However, this process seemed to be present above all in younger adults; on this regard, we can hypothesize that digital natives would be more facilitated to generatively exploit the benefits of the positive re-thinking through a remote modality that requires familiarity with new technologies. This evidence opens up two future directions for research and intervention: first, an in-depth exploration of the causal paths that link the intra-individual process of positive re-thinking during pandemic to prosocial behaviors and caring toward society; further, the need to implement digital modalities to foster civic engagement in older adults, in order not to waste a potential triggering factor for the development of communities. This consideration appears particularly salient if we consider the fourth emerged factor, called “Perception of online social connectedness”, that refers to the extent to which individuals are inclined to perceive social connectedness and support through the online social media. It captures a central process that characterizes the extant pandemic, consisting in the opportunity provided by new digital technologies to maintain social relationships, using of social networks and participating in online communities. This factor was related to high levels of disposition toward civic engagement, as well as - limited to older adults - it showed a positive association with external health locus of control. On the one hand, feeling to be connected with online users could make individuals more inclined to use the same technologies to act in a prosocial way towards others, in a sort of a mutual exchange. On the other hand, older adults may have less familiarity with digital media, and lean on it in a unidirectional way, perhaps obtaining care and comfort but in the direction of amplifying the belief that their health depends on others. Once again, these results highlight that an important effort must be made by social workers and public institutions in making social media and new technologies more accessible to older audience. Such accessibility must concern not only the physical possibility of using devices (i.e., by overcoming economic barriers and problems related to the coverage of the Internet network) but also the competence to use them in an agentic and proactive way, as means for maintaining social connectedness and activating mutual social support. In fact, recent evidences related to the extant pandemic have demonstrated that the quality of social interactions (i.e., in terms of perceived social support) plays a role in promoting resilience and in buffering the association between worry and psychological health (Szkody et al., 2020; Zhang et al., 2021), and the degree of social interactions (defined as the number of individuals that people communicated with during the lockdown) was inversely related to levels of stress and worry (Nitschke et al., 2020), suggesting that social contacts - even if at a distance - can be an important protection factor against the onset of maladaptive outcomes.

The present results must be viewed in light of some limitations. First, the convenience sample used here is highly gender-biased, and therefore the male component of the population may be under-represented. Furthermore, online recruitment could have affected the generalizability of the results, especially with reference to adult population that does not have access or does not habitually use social media; moreover, as for participants in the present study, we have not information about the quantitative and qualitative use they make of social media. Additionally, the research was conducted in a single cultural context - the Italian one - and therefore the generalizability of the results to other Western and non-Western cultures should be investigated. Further, the cross-sectional nature of the present study prevented us from causal conclusion, and future longitudinal studies are needed to confirm the causal links that we hypothesized here. We are also aware that present results refer to the initial validation process of the FPLQ, and the clinical usage of the questionnaire remains to be in-depth explored. For instance, the internal consistency reliabilities for the FPLQ subscales are acceptable for research, but they are questionable for a clinical use that orients specific and personalized interventions. Lastly, it is to note that the emerged factor structure did not concur with the factors identified in our theoretical model; this suggests that the FPLQ assesses specific cognitions and related feelings that are strictly linked to the experience of pandemic, rather than trait-like styles. For instance, within the pool of items originally developed to assess social connectedness, those related to online interactions and relationships emerged as a separate factor, indicating that the component of perceiving online social support could constitute a specific process of the extant pandemic worth of in-depth attention. Moreover, we developed a theoretical worry dimension including aspects related to health, daily routines, and work/economic aspects; actually, exploratory and confirmatory factor analyses identified a specific factor related to health worry. A specific scale focused on health that emerged from a more comprehensive questionnaire whose psychometric properties have been the focus of a specific study could be an important strength, since several recent research on the extant pandemic used single measures on fear about developing infection or infecting others (e.g., Boyraz et al., 2020; Grossman et al., 2021) on the basis of ad-hoc created items, or derived from other broader instruments. Nevertheless, items of the FPLQ related to work/economic worries have failed to emerge both as a specific factor and as items pertaining to a broader factor, and this needs reflection for further development of the questionnaire. However, despite these limitations, the present contribution showed that the FPLQ has promising psychometric properties and that it can be applied to increase the theoretical knowledge related to the psychological implications of the pandemic. Moreover, stimulated by research indicating the need to explore specific psychological processes of specific subgroups (Varga et al., 2021), we highlighted the moderation role of both gender and age, further underlining the need to foresee some tailored actions according to age and gender. At the present, we are not in a national lockdown condition anymore; nevertheless, the FPLQ can be useful for those situations in which individuals are subjected to restrictive quarantine measures due to contagions or close contacts with infected people. Moreover, this questionnaire could be applied in the context of other conditions that could require a forced isolation from others, in order to explore individual differences in adaptive and maladaptive processes in front of the situation.

## Data Availability

Dataset is available on request.
